# The use of contracts to implement and manage healthy vending: best practice recommendations for effective and sustained interventions

**DOI:** 10.1186/s12889-024-20771-8

**Published:** 2024-11-20

**Authors:** Jane Dancey, Belinda Reeve, Alexandra Jones, Julie Brimblecombe

**Affiliations:** 1 Department of Nutrition, Dietetics and Food, Monash University, Melbourne, Australia; 2 The University of Sydney Law School, Sydney, Australia; 3 Food Division, The George Institute for Global Health, Sydney, Australia

**Keywords:** Healthy food, Private regulation, Contracts, Vending, Governance, Best practice

## Abstract

**Background:**

Contracts can be an effective lever to implement and manage health-enabling food retail environments. However, guidance for the effective use of contracts in food retail settings is limited. The use of contracts to create healthy food *vending* environments is one area where policy attention has been focussed in high income countries. We applied a public health regulatory framework to publicly available guidance documents on healthy vending to develop best practice recommendations for using contracts to create healthy food vending environments.

**Methods:**

Document analysis involved i) snowball sampling to identify eligible publicly available healthy vending guidance documents from an identified seed paper; ii) application of a public health regulatory framework to extract data across three domains of form, substance and governance of healthy vending initiatives; and iii) synthesis of data to form best practice recommendations. Eligible documents were those aimed at implementing healthier vending; published from 2000 onwards; accessible online; and included recommendations beyond nutrition standards alone, including a reference to at least one regulatory governance process (administration, implementation, monitoring, enforcement or review).

**Results:**

Twelve of 92 documents identified were eligible and all were from the United States (US). All noted that products need to comply with nutrition standards. Other aspects of regulatory substance (i.e., pricing, promotion, placement, labelling and contract length) were less well considered as were elements of regulatory governance (regulatory rules, administration, implementation, monitoring, enforcement and review). Our adapted framework covers three regulatory domains with nine components, and a further 20 recommendations for best practice application in healthy vending.

**Conclusions:**

To be effective, contracts used to manage healthy food vending should include more than the nutrition standards for healthy food and drinks. Clearly stating the contract objectives, operative terms and conditions, and defining responsibilities for monitoring, review and enforcement within the contract, in addition to the nutrition standards, will assist practitioners in creating effective and sustained contract-based initiatives aimed at improving the healthiness of vending, or potentially other food retail environments.

**Supplementary Information:**

The online version contains supplementary material available at 10.1186/s12889-024-20771-8.

## Background

Global food systems are dominated by unhealthy food and drinks products and their consumption increases the risk of developing non-communicable diseases (NCDs) such as heart disease, stroke, diabetes and cancer [1, 2]. To enable the consumption of healthier food and drinks, evidence supports a shift from policies that focus solely on individual responsibility for healthy food and drink choices to those that support the creation of healthier food environments [3–5]. The World Health Organisation (WHO) has set out a number of food environment-related policy options to decrease mortality from NCDs [6, 7]. These policy options involve significant changes to the food system, including the food retail environment. In this paper, we examine healthy food and drink vending, as an example of how a transition to a healthier food retail environment can be made. Specifically, we examine the use of contracts to implement and sustain healthy changes in vending.

Heeding the WHO’s call, some countries have introduced government regulation to create healthier food environments in, for example, the form of taxes on high sugar drinks, marketing restrictions on unhealthy food and drink products, and geographic zoning to prevent the environments surrounding schools from being dominated by fast food outlets [8–12]. Government (or public) regulation has also been used to create healthier food *retail* environments in certain settings specifically, for example by mandating food and menu labelling and creating policies to restrict the sale of unhealthy food and drinks [13–15]. Non-government actors, however, also use regulation to create healthier food retail environments such as the food industry’s self-regulatory reformulation initiatives to lower salt or sugar in food and drinks, and the use of regulation in the form of leases, contracts and policies to create healthier food retail outlets, canteens and vending by other, non-food industries (such as health services, universities and sports centres) [16–19]. In this research we draw on literature and concepts from the field of regulatory studies to conceptualise the use of contracts between a property owner and a provider to enforce nutrition standards in food and drink vending as a form of private regulation (defined here as forms of regulation that are created and implemented by non-government entities, including universities and hospitals, for example), and one that has received less attention than industry-led self-regulation, or public–private partnerships between industry and government to improve diet-related health [18]. Contracts could potentially be an important public health tool. Unlike industry self-regulation, contracts concerned with improving the food retail environment involve one party (such as a hospital or university) requiring another party (such as a food retailer operating within that organisation) to sell healthier food and drinks, through the use of contractual terms. The binding nature of contracts and their scope for meaningful enforcement processes where a party breaches conditions, is something that is often lacking in self-regulatory schemes [20]. However, there is little documented in the literature on the use of contracts to create healthy food retail initiatives and even less regarding the elements that might contribute to an effective contract [21].

Private regulation may act as a catalyst for positive shifts in the health-enabling policies and practices of food retail operators, particularly in the context where there is a lack of political will for public regulation to create healthier food environments, such as through the use of sugar sweetened beverage (SSB) taxes and junk food marketing restrictions [9, 22]. Whilst large-scale changes to the food environment are unlikely to occur without public regulation, we explore the use of private regulation by organisations and individuals as an exemplar for change, or as an example of an implementation strategy that could work in conjunction with government-led regulation [23].

This paper examines healthy vending initiatives, using contracts, as a form of private regulation, between an organisation and a vending service provider rather than private self-regulatory initiatives developed by the food industry, which arguably seek to avert further public or government regulation and have been the subject of significant criticism of the extent to which they have achieved public health outcomes [9].

Vending differs from the more traditional ‘bricks and mortar’ food retail setting (cafes, restaurants, supermarkets) in that vending machines are usually distributed through a contractual arrangement between an organisation and the vendor, with the organisation receiving a negotiated percentage of sales. Contracts provide a potential mechanism for organisations to stipulate the type of food and drinks offered (including nutritional criteria); their placement and price within the machines; the location of machines across the physical environment; the marketing and promotions allowed; and the sharing of sales data. There has been moderate success with the use of contracts to transition away from vending machines stocking unhealthy, ultra-processed food and drinks in large and diverse settings, such as schools, hospitals/health services, workplaces and universities, to vending machines that stock healthier food and drink options [15, 17, 24]. However, in a systematic scoping review of the types and governance of private regulatory measures used to create healthy food retail environments, we found published information regarding the implementation of private regulation but very little detail about the other governance mechanisms of monitoring, review and enforcement, which evidence suggests, are important for effective implementation and impact [21, 25, 26].

The paper reports on how contracts should be implemented or governed. Research from the fields of regulatory studies and public health governance offer important insights into how to design private regulation to be most effective, including adequate mechanisms for administration, monitoring, enforcement and review [25–27]. Transparent monitoring and reporting mechanisms allow internal and external stakeholders to evaluate the performance of the scheme in achieving its objectives, and enables enforcement action for mandatory schemes. Monitoring also enables improvements in compliance and the design of the scheme itself, as well as enhancement of the transparency and accountability of private regulation [28]. Enforcement and review mechanisms are similarly important to enhance the transparency, accountability and credibility of private regulation, to enable continuous improvement and deter non-compliance [28]. Accordingly, this paper develops best practice recommendations for the governance of healthy vending initiatives, using contracts, as well as their substantive content.

For those looking to implement healthy vending, there are a variety of ‘how to guides’ publicly available online [29, 30]. These guidance documents vary in their breadth, covering matters ranging from the nutrition standards that guide the nutritional composition of the products being sold, to building support for implementation and contract arrangements. Guidance on the full range of governance issues, including monitoring, review and enforcement, in addition to implementation and nutrition criteria, should enhance the effectiveness of healthy food vending initiatives given their importance to the effectiveness of regulation. Without such best practice mechanisms, implementation is difficult to sustain and the ability to achieve the desired health outcomes can be undermined. Accordingly, the aim of this paper is to synthesise guidance on healthy vending against an established public health regulatory framework to develop best practice recommendations for effective healthy vending contract implementation by practitioners and/or policy makers [25, 27].

## Methods

**Fig. 1:**
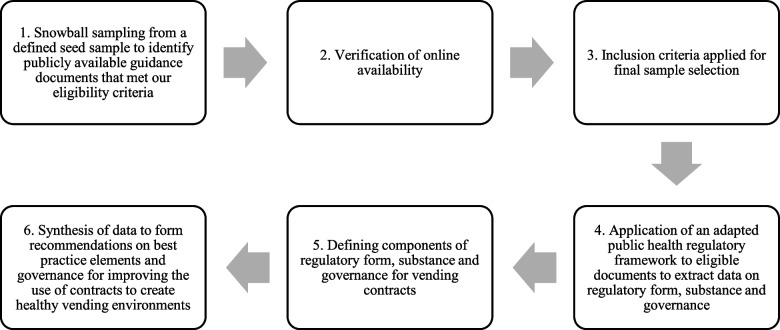
Six stages of document analysis

Document analysis involved six stages (Fig. 1):

i) snowball sampling from a defined seed sample to identify publicly available guidance documents that met our eligibility criteria;
ii) verification of online availability;
iii) inclusion criteria applied for final sample selection;
iv) application of an adapted public health regulatory framework to eligible documents to extract data on regulatory form, substance and governance;
v) defining components of regulatory form, substance and governance for vending contracts; and
vi) synthesis of data to form recommendations on best practice elements and governance for improving the use of contracts to create healthy vending environments.

### Sampling to identify guidance documents

First, a seed document was identified. This was a recently published article by Green et al. that examined the facilitators and barriers to developing, implementing and evaluating healthy vending policies [31]. This article recommended four online guidance documents to use when developing and implementing healthy vending initiatives [29, 32–34].

### Verification of online availability

The hyperlinks for these four online guides were then tested. However two had expired so the first author (JD) searched the hyperlink websites (Center for Science in Public Interest and Public Health Law Centre at William Mitchell College of Law) for new hyperlinks and then verified that these matched the intended guides with the seed sample’s lead author (Sarah Green) via email communication [31]. A fifth document was included in the seed sample as one of the hyperlinks (ChangeLab Solutions) lead to two separate documents: a PDF guide and an MS Word document with separate guidance for healthy vending [29, 32–35]. The next step involved the import of all references (citations) contained in each of the five seed sample documents into a MS Excel spreadsheet.

The hyperlink (where provided) for each reference in the snowballed sample was then tested and if it did not link to the cited document, the website was searched for the document title. To finalise the sample, if the document was found on the corresponding website the new hyperlink was added to the MS Excel spreadsheet and if it was not found, it was noted that the document was no longer accessible online and these documents were excluded. Other references that were found not to be publicly available online were also excluded as per our inclusion criteria for online accessibility.

### Inclusion criteria for final sample

Inclusion criteria applied to all identified documents and required that the document was 1.) published from 2000 onwards; 2.) aimed at implementing healthier vending; 3.) accessible online; 4.) made recommendations beyond nutrition standards alone including a reference to at least one regulatory governance process (such as administration, implementation, monitoring, enforcement or review). Based on these criteria, all guidance documents were focussed on the implementation of healthier vending.

Once the inclusion criteria were applied, one of the seed sample documents was excluded [34] on the basis that it compares nutrition standards only. However, the references included in that document were still included in the snowballing process. Application of an adapted public health regulatory framework.

Our data extraction template was informed by a public health regulatory framework designed to facilitate effective, transparent and accountable regulatory design, in the context of healthy vending [27]. This framework by Reeve and Magnusson is based on the literature from regulatory studies and public health, and outlines three regulatory ‘domains’ and their components that can be used to evaluate and strengthen regulatory design [36]. Reeve and Magnusson’s framework has been further adapted by others and we used terminology from these frameworks, in addition to the original [25, 37]. We adapted the components used in each of the framework’s domains of regulatory form, regulatory substance and regulatory governance, and applied them to the healthy vending context.

### The three domains of healthy vending contracts

#### Regulatory form

Adapting the framework to the healthy vending context, regulatory form, includes two components: the regulatory framework which examines where in the local/national/global legal and policy context the regulation fits; and whether the regulation recommends mandatory or voluntary implementation.

#### Regulatory substance

Regulatory substance includes two components 1) clearly stated and measurable objectives by which the initiative can be assessed; and 2.) clearly defined operative (key) terms and conditions [36]. Adapting these components to the healthy vending context, we considered that recommendations to include clear and measurable objectives could be described in the policy and contract guidance documents, and/or, the policy documents could provide examples of clearly stated and/or measurable objectives – both of which could be instructive for best practice. For example, a policy document may contain a clear and measurable objective, such as ‘to increase the availability of healthy food and drinks in vending’ but would not provide guidance on how to write one. The operative terms and conditions of healthy vending contracts related to the types of *products* allowed in vending machines (including the type of nutrition standards recommended), the *placement* of products within the machines, the *promotion* of healthy and unhealthy products within and outside the machines, including the vending machine ‘skin’ or ‘wrap’, and the *pricing* of products sold in the machines. We also extracted information on identification (labelling) of healthier products in the vending machines and any guidance around optimal contract length.

#### Regulatory governance

Regulatory governance relates to the drafting of the rules and scheme design including accountability and transparency in the design; administration by an identified (and/or independent if applicable) body which can monitor and enforce the initiative; implementation details where key steps are set out as contractual terms; monitoring including the collection of baseline data, setting process and outcome indicators; enforcement including soft and hard measures to encourage compliance; and a review process to ensure a structured, regular review of the initiative including an analysis of data obtained through monitoring [36].

### Synthesis of data to form best practice recommendations

Each guidance document was reviewed and all text relevant to each of the domains and their specific components was extracted and summarised to retain original meaning. Where no text was identified that matched with a framework domain and/or its specific component, this was recorded as ‘not described’. When guidance documents referenced language specific to their US jurisdiction, we moderated that language to apply to a broader international audience on the advice of our co-authors and legal/regulatory scholars (BR and AJ). In addition to details on regulatory form, substance and governance, document characteristics including: document name, country, date of publication, author, type of guidance document, target and type of vending described, were also extracted.

Guided by the recommendations in the three domain and nine component framework composed by Reeve and Magnusson and adapted by others, we examined our extracted data to identify recommendations and best practice examples to populate our adapted framework [25, 27, 37].

## Results

A total of 92 documents were identified (five from the seed sample, and 87 from the references). Twelve documents met the eligibility criteria and remained for extraction. Of the 80 documents excluded, 40 were not accessible online; 29 were not aimed at implementing healthier vending; 6 did not make recommendations beyond nutrition standards; 1 was published prior to 2000; and 4 were duplicates.

### Characteristics of documents identified

Table 1 outlines the characteristics of the documents identified. Of the twelve healthy vending documents identified, four were implementation guides [38–41], three were policy guides, [30, 32, 33] two were contract guides [35, 42], two were both policy and implementation guides [29, 43], and one was a local government policy document [44]. All documents referred to healthy vending, although some limited their scope to government-owned vending [39, 43] and one document referred specifically to the school vending environment [42]. Documents were all published in the US between 2006–2022. Only one of the 12 documents referenced each component included in the three domains of regulatory form, substance and governance [29].

**Table 1:** Characteristics and substantive content of contracts in healthy vending

*Document characteristics*					*Regulatory substance*						
***Document name, jurisdiction, year published***	***Who developed the document***	***Type of guidance***	***Who is expected to implement and comply?***	***Type of vending described***	***Regulatory Objective***	***Nutrition standards***	***Labelling***	***Placement***	***Promotion***	***Pricing***	***Contract length***
Key Components of Food Procurement and Vending Policies, Drafting an Effective Policy, USA, 2015 [32]	Public Health Law Centre at William Mitchell College of Law	Healthy food and drink procurement and vending policy guidance	Government and private organisations who want to write effective healthy vending and procurement policies	Snack and beverage vending, snack stands and cafeterias	Guidance to include a purpose or goal statement that can include an educational opportunity	Include evidence-based nutrition standards in contracts	Not described	Prioritise placement of products meeting nutrition standards	Promote only products that meet nutrition standards. Promotional space on machines to only promote products that meet nutrition standards	Ensure products that meet nutrition standards are affordable and lower in price than products that do not. Offer discounts and promotions on products that meet nutrition standards	Not described
Model Beverage and Food Vending Machine Standards, USA, 2012 [41]	A workgroup of approximately 40 NANA members with expertise in food and nutrition	Model healthy vending standards and implementation guidance	Municipal, state, and federal government or public property vending machines. Can be used by hospitals, private workplaces, and others	Food and beverage vending	Not described	Evidence-based nutrition standards for food and beverages are detailed	Recommends point of purchase calorie labelling	Prioritise placement of products meeting nutrition standards (e.g., at eye level)	Promotional space on machines used only for products that meet nutrition standards	Competitive pricing for products that meet nutrition standards	Not described
Tips for Better Vending, USA, 2013 [30]	Public Health Law Centre at William Mitchell College of Law	Vending policy guidance	Municipalities and facility operators	Food and beverage vending	Not described	Source relevant and evidence-based nutrition standards to inform policy	Brief mention of use of labelling system (nfd) and reference in ‘Additional Resources’	Prioritise placement of products meeting nutrition standards (e.g., at eye level)	Water brands featured on machines instead of sugary drinks	Products that meet nutrition standards must be affordable and lower in price than products that do not meet nutrition standards	Not described
Healthy Food and Beverage Toolkit, USA, 2022 [33]	American Heart Association (AHA)	Policy guidance	Organisations/workplaces	Workplaces in general and snack and beverage vending	Not described	Reference to AHA’s own ‘Healthy Eating Recommendations and Nutrition Standards’	Recommends point or purchase calorie, sodium and sugar labelling	Prioritise placement of products meeting nutrition standards (e.g., at eye level)	Promotions, signage and machines only promote products that meet nutrition standards	Products meeting nutrition standards priced competitively or discounted	Not described
Making Change: a guide to healthier vending for municipalities USA, 2012 [29]	ChangeLab Solutions.^a^	Healthy vending policy guidance and policy implementation guidance. Can be used in conjunction with ChangeLabs Model Healthy Municipal Snack and Beverage Vending Agreement	Municipalities looking to develop a healthy vending policy to improve the food environment for people working for, visiting, and being served by local government agencies	All vending	Guidance to define what you want to change and where, and to be strategic, specific and realistic	Reference to example standards and recommendation to base standards on the Dietary Guidelines for Americans	Recommends point of purchase calorie labelling	Prioritise placement of products meeting nutrition standards (e.g., at eye level)	Promote only products that meet nutrition standards and promotional space on machines used only for products that meet nutrition standards	Products meeting nutrition standards priced competitively (less or same as unhealthy products)	Keep contract term short
Model Healthy Municipal *Snack and Beverage* Vending Agreement, USA, 2012 [35]	ChangeLab Solutions	Healthy vending contract guidance. How to write a good healthy vending contract	Nutrition advocates and municipalities wanting to implement healthy vending contracts	Snack and beverage vending	Not described	Guidance to include ‘permitted’ snack and beverage products in vending contract	Recommends point of purchase calorie labelling	Healthiest options in highest selling positions	No promotion by the vendor. Only promotion of products that meet nutrition standards or municipal advertising	Establish lower prices for healthy options	Keep contract term short and refer to state and local laws governing contract terms. Long terms are anti-competitive
Model Healthy *Beverage* Vending Agreement, USA, 2012 [42]	National Policy and Legal Analysis Network to Prevent Childhood Obesity (NPLAN) and ChangeLab Solutions	Healthy vending contract guidance	Nutrition advocates and school districts using the contracting process to achieve healthy vending	Beverage vending in schools	Not described	Reference to a ‘permitted’ healthy beverage policy specifications sheet which lists beverages allowed for sale	Not described	Not described	Vendor has no promotion or advertising rights. Any advertising approved by the District	Not described	5-year maximum contract term
Good Choice. Guidelines for Successful Healthy Vending Machines in Alabama, USA, 2011 [38]	Alabama Department of Public Health in collaboration with the Alabama Department of Rehabilitation Services	Healthy vending implementation guidance	Designed for state agencies but can be adopted and utilized by any place of business	Food and beverage vending in state owned/operated buildings and participating businesses	Document’s objective is to increase access to healthy food and beverages and reduce or eliminate availability of unhealthy food in public service venues	An approved snack list is included and a hyperlink to Alabama healthy vending machine website	‘Good Choice’ sticker to denote healthier items	Products meeting nutrition standards should be grouped; in centre or far left; and stocked consistently	‘Good Choice’ promotional materials (stickers, posters, brochures, table tents), and guidance to promote the initiative	Price of healthy items not set higher than regular version of similar product	Not described
Understanding Healthy Procurement: Using Government’s Purchasing Power to Increase Access to Healthy Food, USA, 2011 [43]	NPLAN	Government (local and State) healthy procurement policy guidance and procurement policy implementation guidance	State and local governments wanting to adopt healthier procurement policies in vending	An explanatory guide to healthy government procurement in both 1.) institutionalised food service (jails, public hospitals and schools) and 2.) vending machines, cafeterias, concession stands and other retail outlets on government property	Not described	Guidance to include health and nutrition standards in the contract	Recommends point of purchase calorie labelling	Not described	Not described	Not described	Not described
Health and Sustainability Guidelines for Federal Concessions and Vending Operations, USA, 2012 [39]	The US Department of Health and Human Services (HHS) and the US General Services Administration (GSA)	Implementation guidance	The guidelines apply to all food service concession operations and vending machines managed by Health and Human Services (HHS) and General Services Administration (GSA). They may also be applied at sponsored or co-sponsored conferences and events onsite and offsite, as deemed appropriate	The guide applies to all federal concessions and vending operations	Not described	Standards for packaged food and beverages included in the document	Recommends point of purchase calorie labelling	Not described	Not described	Not described	Not described
King County Healthy Vending Implementation Toolkit, USA, 2011 [40]	Public Health: Seattle and King County	Implementation guide	The toolkit was designed for "Worksite wellness programs, Schools, Youth programs, Government buildings, Hospitals and other healthcare delivery settings, Social service organizations, Community-based organizations, Family-oriented museums and entertainment venues"	All vending	Guidance to set goals and objectives, how to develop them, and examples given	King County Healthy Vending Guidelines (which contain nutrition standards) included as an appendix	Recommends labelling healthy items with stickers or product pushers	Prioritise placement of products meeting nutrition standards (e.g., at eye level) and grouped together	Limit advertising on machines to the healthiest products	Guidance to work with vendor to make healthier items less expensive	Not described
Healthy Choice Options in Vending Machines on County Property, USA, 2006 [44]	San Diego County Board of Supervisors	Policy document	Vending machines on San Diego County Property	Snack, beverage and entrée vending	Document’s objective is to establish guidelines to provide healthy-choice options in vending machines on Country property	Standards for beverages, snacks and entrees given	Not described	Not described	Not described	Products meeting nutrition standards are to be priced comparatively	Not described

*Nfd* no further details

^a^ChangeLab Solutions is a non-profit organization that provides legal information on matters relating to public health

### Application of best practice public-health regulatory framework

#### Regulatory form

##### Regulatory framework

Three documents (25%) specifically noted the need for healthy vending initiatives to be coherent with the local legal and policy context, for example, that any vending contract should comply with State/local laws and organisational policy as shown in Table 1 [29, 35, 40].

Only one document (8%) recommended that healthy vending initiatives be made mandatory to strengthen effectiveness [29]. Another was co-authored by, and targeted at, two government departments and for one department the healthy vending guidance was mandatory, and for the other department it was ‘strongly encouraged’ but not mandatory [39]. The three documents (25%) that referred specifically to procurement or contractual guidance, made no mention of mandatory implementation, although contracts could be seen as binding as contractual terms are enforceable if breached by either party [35, 42, 43]. Five documents (42%) made no mention of voluntary or mandatory status in the vending guidance [30, 32, 33, 41].

#### Regulatory substance

Table 1 also provides an overview of the data extracted on regulatory substance, including regulatory objectives and the operative terms and conditions.

##### Regulatory objective

Five documents (42%) had a clearly stated and/or measurable objective or recommended the inclusion of a clearly stated and/or measurable objective. For example, Alabama’s Good Choice Guideline’s objective was both clear and measurable: “to increase access to healthy foods and beverages and reduce or eliminate the availability of calorie dense, nutrient poor foods in public service venues” [38]. Three guidance documents recommended that users clearly state an objective and gave guidance on how to do so, such as the King County Healthy Vending Implementation Toolkit which provided a section on setting goals and objectives, and gave examples [29, 32, 40].

##### Operative terms and conditions

•Product

All documents noted that the food and drink (the product) in the machine needed to comply with nutrition standards. Eight documents (67%) recommended either the use of evidence-based nutrition standards and three (25%) recommended or included a list of pre-determined products suitable for vending machines. One document (8%) referenced nutrition standards and also included a list of suitable products as an appendix [40].

- Price

Nine of the twelve (75%) documents described the need for healthier products to be priced lower, or competitively with less healthy products.

- Promotion

Seven documents (58%) described the need to promote only products that meet the nutrition standards being adopted [29, 32, 33, 35, 38, 40, 41]. Two documents (17%) described a requirement that the vending operator had no rights to promotion or advertising [35, 42]. Three documents (25%) did not mention the importance of promotional considerations [39, 43, 44]. One document (8%) gave specific instruction for the outside of the vending machine to display only water brands instead of sugary drinks [30].

- Placement

Eight documents (67%) described how the healthiest products should be in the most prominent, highest selling positions. Four documents (33%) made no mention of where products should be placed in vending machines [39, 42–44].

- Labelling

Eight documents (67%) recommended that products should be labelled at the point of purchase. One document noted that there should be a ‘labelling system’ but provided no additional context [30]. Three documents (33%) made no mention of labelling of healthier products in vending machines [32, 42, 44].

- Contract length

Three documents (25%) stated that contract length should be time-limited [29, 35, 42]. These three documents were all produced by ChangeLab Solutions, a US-based not for profit organization that uses the tools of law and policy to advance health equity. The guidance ranged from general advice about keeping the contract length short and deferring to local laws, to more specific guidance to not to exceed 5 years contract length.

#### Regulatory governance

Table 2 describes the elements of regulatory governance identified in our sample, including the drafting of rules and the scheme design, administration, implementation, monitoring, enforcement and review processes. In this study one third of the documents noted who should administer the contract, 67% of documents commented on implementation and monitoring processes, 42% described an enforcement process and 67% noted the need for a review process.

**Table 2:** Regulatory form and governance in healthy vending

	***Regulatory form***		***Regulatory governance***					
***Document, jurisdiction, year published***	***Regulatory framework***	***Mandatory policy recommendation***	***Guideline Development***	***Administration***	***Implementation***	***Monitoring***	***Enforcement***	***Review***
Key Components of Food Procurement and Vending Policies, Drafting an Effective Policy, USA, 2015 [32]	Reference to consistency between internal policy and contracts	Not described	Not described	Guidance to identify who enforces the policy	Guidance to include how, by when and who will implement for policy and contracts	Guidance to include record keeping and reporting by vending companies in contract. Recommends a pre-implementation assessment for baseline data. Guidance on conducting a baseline assessment to track changes once policy in place	Guidance to include enforcement provisions and clauses to address compliance problems in contracts. Guidance to identify the specific person or department responsible for enforcement	Guidance on importance of evaluation including tracking toward goals, changes in sales and purchasing patterns; vendor compliance; communicating results to management and community; and seeking feedback to strengthen the policy
Model Beverage and Food Vending Machine Standards, USA, 2012 [41]	Not described	Not described	A working group of 40 people collaborated on this document	Not described	Guidance on phased implementation and setting percentage healthy and time-specific implementation (50% healthy by 1 year)	Not described	Not described	Not described
Tips for Better Vending, USA, 2013 [30]	Not described	Not described	Not described	Not described	Not described	Not described	Not described	Guidance to ensure a process for evaluation is in place
American Heart Association Healthy Food and Beverage Toolkit, USA, 2022 [33]	Not described	Not described	Guidance to work with internal and external stakeholders (human resources, facilities operations, food and beverage vendors, registered dietitian, unions, insurance provider and employee health specialist)	Not described	Detailed implementation guidance including a ‘gradual’ approach	Guidance to ‘monitor’ but no further detail	Not described	Guidance to evaluate and communicate results
Making Change, A Guide to Healthier vending for municipalities, USA, 2012 [29]	Understand impact of state and local laws. Consult with legal counsel to adapt the guidance to relevant municipality	Recommendation from public health and policy change perspective to make policy mandatory	Guidance to work with internal and external partners (key employees, community members, purchasing and other experts, government agency and other department leaders, champions, food experts, industry and other allies)	Guidance to designate staff to monitor and review regularly	Detailed implementation guidance including creation of implementation goals, strategies, timelines and ongoing stakeholder engagement	Guidance to monitor and report back regularly (quarterly or 6 monthly)	Guidance to include strong enforcement provisions	Guidance to conduct an annual review to evaluate and revise guidelines as needed
Model Healthy Municipal Snack and Beverage Vending Agreement, USA, 2012 [35]	Understand impact of state and local laws. Consult with legal counsel to adapt the guidance to relevant municipality	Not described, but guidance is for contracts which are binding	Not described	Not described	Not described	Guidance to include clauses that vendor provide accurate monthly sales reports (by facility, machine and product)	Guidance to include a compliance statement and a section in the contract outlining termination if breach of contract for non-compliance with nutrition standards	Guidance to include a ‘revisions of policy’ arrangement in contracts to allow for changes or updates to occur
Model Healthy Beverage Vending Agreement, USA, 2012 [42]	Broad discussion in Recitals section regarding who has authority	Not described, but guidance is for contracts which are binding	Not described	Not described	Not described	Guidance to include monthly financial reporting	Guidance to include termination of contract if there is a failure to comply with nutrition standards	Guidance to conduct quarterly meetings with the vendor to review sales, nutrition, efficiency and communication
Good Choice. Guidelines for Successful Healthy Vending Machines in Alabama, USA, 2011 [38]	Not described	Guidelines are highly recommended but not mandatory	Not described	Not described	Discussion of workplace champions to implement and maintain	Not described	Not described	Not described
Understanding Healthy Procurement: Using Government’s Purchasing Power to Increase Access to Healthy Food, USA, 2011 [43]	Only in reference to the use of local suppliers (consult with local government attorney)	Not described but reference to contracts which are binding	Guidance to involve parents, students and community members	Not described	Broad implementation guidance starting with contacting policy makers, attending public meetings, obtaining copies of vending contracts and then once policy adopted, ensuring contracts comply	Broad guidance to include a process for monitoring	Guidance to include penalties (including financial penalties) in the contract for non-compliance There is also an example of an incentive used to encourage compliance by providing discounted permits to vendors who comply with the healthy food policy	Not described
Health and Sustainability Guidelines for Federal Concessions and Vending Operations, USA, 2011 [39]	Not described	Mandatory for food sold at HHS and strongly encouraged for properties managed by GSA	HHS and GSA worked collaboratively to develop the Guidelines	Not described	Not described	Not described	Not described	Reference to annual assessment of sodium values based on consumer feedback, contractor feasibility and nutrition science, but no further detail on this review process
King County Healthy Vending Implementation Toolkit, USA, 2011 [40]	Consult with purchasing or legal departments	Not described	Not described	Guidance to assign a specific person to monitor and maintain changes	Detailed implementation guidance on planning for change, building a team, gather information, developing a plan, implementing the change and evaluating	Detailed guidance on what to monitor, when, who will monitor and who to report to. Examples given to monitor labelling, pricing, location, signage, sales figures and revenue	Not described	Guidance to conduct evaluations including documenting process and outcomes, monitoring and maintaining changes and evaluating vending usage and sales
County of San Diego, California Board of Supervisors Policy, Healthy Choice Options in Vending Machines on County Property, USA, 2006 [44]	Not described	Reference in policy that vending machines ‘shall’ comply with nutrition standards outlined, indicating mandatory requirement	Not described	Reference to who is responsible for annual review	Not described but there is reference to a separate implementation guide	Monitoring procedures must be in place to meet the nutrition standards but no further detail	Not described	Annual review process by the County is documented in the policy

##### Drafting rules and scheme design (guideline development)

Three documents (25%) noted the need to work with stakeholders when drafting healthy vending initiative documentation [29, 33, 43]. In addition, The National Alliance for Nutrition and Activity (NANA) document had itself been developed in consultation with approximately 40 stakeholders with expertise in food and nutrition [41] and the Health and Sustainability Guidelines for Federal Concessions and Vending Operations was a collaboration between two government agencies (Health and Human Services, and General Services Administration) [39].

##### Administration

Four documents (33%) stated that there should be reference to precisely *who* monitors, enforces or reviews the healthy vending initiative [29, 32, 40, 44].

##### Implementation

Eight documents (67%) commented on implementation processes. Implementation guidance included starting with policy formulation, if no policy was in place and then moving to implementation via vending contracts once the policy had been adopted [32, 43]. Seven documents (58%) provided detailed implementation guidance including planning for change; who, when and how the initiative should be implemented; creating implementation goals, strategies and timelines; keeping stakeholders engaged; and the use of workplace champions [29, 32, 33, 38, 40, 41, 43].

Two documents (17%) recommended a phased or gradual approach to implementation as means to ease the transition to healthy vending [33, 41]. Three documents (25%) did not provide any implementation guidance [30, 35, 42]. Two of those documents related specifically to contract guidance, so guidance on implementation was not within the scope of these documents [35, 42]. One document (8%) did not refer to implementation but referred to a separate implementation guide [44].

##### Monitoring

Eight documents (67%) detailed a form of monitoring ranging from high level to more detailed guidance. High level guidance included the San Diego Board of Supervisors Policy stating that ‘All County Departments shall establish monitoring procedures to ensure that all vending machines located in their respective Departments meet the healthy-choice nutrition standards’. More detailed guidance was given by the King County Healthy Vending Implementation Toolkit describing what to monitor, when, and by whom [40, 44]. For contracts, recommendations were given to include provisions/clauses that the vending operator must provide monthly sales figures (per facility/machine/product) [32, 35, 42]. Four documents (25%) made no mention of monitoring [30, 38, 39, 41].

##### Enforcement

Five documents (42%) described the inclusion of enforcement provisions in vending contracts [29, 32, 35, 42, 43]. Guidance included the insertion of compliance statements in contracts relating to termination of contract or financial penalties for non-compliance with nutrition standards [32, 35, 42]. One document (8%) referred to an incentive to encourage compliance, namely a discounted permit to vendors who comply with the healthy food initiative [39]. Seven documents (58%) made no mention of enforcement [30, 33, 38–41, 44].

##### Review

Nine documents (75%) mentioned the need for a review or evaluation process. The guidance included evaluation of how the healthy vending initiative is tracking towards its goals; changes in sales and purchasing patterns; vendor compliance; communicating results to management and the community; and seeking feedback to strengthen the policy or initiative [32, 40, 42]. Three documents did not mention a review process [38, 41, 43].

#### Synthesis of data to form recommendations on best practice

Only one document referenced all nine components of the three domains therefore best practice elements were drawn from across the guidance documents [29]. Table 3 outlines the best practice elements for healthy food and drink vending that we identified from the guidance documents relating to the three domains of regulatory form, substance and governance and their nine components.

**Table 3:** Best practice recommendations for improving the use of contracts to create healthy vending environments

Component	Recommendation	Best practice application to healthy food and drink vending
***Domain One: Regulatory Form***		
Regulatory framework	The regulatory framework is coherent with the local legal and policy context [25]	The vending contract to comply with state/local laws and organisational policies. Mandatory initiatives overcome suboptimal voluntary implementation and should be considered [25, 35] The contract fits within the local policy context. Advice should be sought from local/organisational legal counsel (if relevant) [29, 35, 40]. Ensure any discussion about contracts has local stakeholder engagement and approval [33]. Contracts will override policy so consistency with local or internal protocols/policy/law is important [40]
***Domain Two: Regulatory Substance***		
Regulatory Objective(s)	Clear and measurable objectives by which success can be measured [27]	The objective of the contract is specific, realistic and based on organisational values ensuring that vendors and readers understand its intention and why it is necessary [29, 32, 38, 40, 44]
Operative terms and conditions	Key terms and conditions are clearly defined. Regulatory rules are sufficiently expansive to achieve the objectives [27]	**Product**: explicit nutrition standards for food and drink to be included, either with reference to clearly defined, evidence-based nutrition standards appropriate to the jurisdiction or as a list of allowable products [29, 30, 32, 33, 35, 38, 40–44]. Reference to nutrition standards may be preferable as it allows ongoing product revisions to occur whereas a fixed list will require a mechanism for contractual revisions **Price**: criteria included in contract to ensure products meeting the nutrition standards are a) affordable and b) priced favourably compared to other products [29, 30, 32, 33, 35, 38, 40, 41, 44] **Promotion**: criteria included to ensure items meeting the nutrition standards are promoted with agreed signs, stickers, and shelf tags. Criteria included to ensure items that fail to meet nutrition standards are NOT promoted within or outside the vending machine. Contracts to include criteria for vending ‘skins/wraps’^#^ to promote only products that meet the nutrition standards or the organisation’s branding (not the vending company’s logo or products that fail to meet the nutrition standards) [29, 30, 32, 33, 35, 38, 40–42] **Placement**: criteria included to ensure products meeting the nutrition standards to be placed in prominent, high selling, eye-level positions. Criteria also included to ensure products that do not meet nutrition standards are not to be placed in high-selling positions [29, 30, 32, 33, 35, 38, 40, 41] **Labelling requirements:** include criteria to ensure point of purchase labelling of healthy products is clear and conspicuous. Contracts can include product labelling requirements including exact location of label (proximal to the product), font size and colour [33, 35, 38–41, 43] **Contract length** to have a clearly defined start and end date and to be kept short (less than 5 years) to allow for market competitive conditions; a chance for review toward the end of the term and to maintain support and ownership within the organisation it was implemented [29, 35, 42]. Contract length may be governed by local or state laws in some jurisdictions
***Domain Three: Regulatory governance***		
Drafting regulatory rules and scheme design	Transparency and accountability mechanisms are incorporated into regulatory measures from their inception [27]	The organisation retains responsibility for the contract. Contracts (and supporting policies) are developed with broad and representative stakeholder input [41]. This may include employees, community members, human resources (or staff wellbeing), facilities management, procurement, nutrition experts, unions, food and beverage vendors and health insurance providers and other allies [29, 33]
Administration	Administration by an independent body which monitors and enforces the initiative [25, 27]	Identify who owns the specific roles required (manage the contract: assign who monitors, enforces and reviews) [29, 32, 40]
Implementation	Key steps to implementation are set out as contractual terms	If no policy exists prior to contract initiation, start by drafting a healthy vending (or food or procurement) policy then once the policy has been adopted, ensure vending contracts are consistent with policy [43] If a policy is already adopted, implementation timelines should be incorporated into vendor contracts with expectations around transition times clearly documented [32, 33, 41] Internal implementation guidance should include stakeholder engagement (including leadership), selecting champions; promotion of the initiative, where to place machines, ongoing staff communication, monitoring, enforcement and review processes [29, 32, 38, 40]
Monitoring	Monitoring includes collecting baseline data, setting process and outcome indicators and timeframes for their achievement and ongoing data collection. Monitoring results are publicly available allowing for scrutiny and feedback that facilitates improvements in the initiative’s operation [25, 27]	Sales data provided by vending businesses to be included in contract terms. As a minimum, the vendor should be required to provide electronic total monthly sales figures and per location/per machine/per item. The inclusion of data transfer from vending operators for monitoring purposes can be included as an enforcement provision [32, 35] Assign a specific role within the organisation to monitor machines quarterly [29] for compliance with nutrition standards [44]; adequate stocking; correct labelling; placement (prominent positions), pricing (if pricing is fixed); and promotion standards. Ensure non-compliance is communicated back to the vending business for prompt attention [40]. Monitoring reports to be communicated back to the team responsible for implementation and to the vending business quarterly [29, 42]
Enforcement	A wide range of enforcement options exist, including incentives to encourage high levels of compliance, ‘soft’ enforcement measures such as persuasion, and more punitive measures for instances of serious or persistent non-compliance [27]	Enforcement provisions (consequences for non-compliance) should be strong and incorporated into the contract [29, 32, 43]. Consequences can be monetary penalties (fines) or incentives [43]. Provisions for termination of contract may be included for breaches of matters that are fundamental to the contract [35, 42, 43]. Identify the specific person or department responsible for enforcement [32]
Review	Structured, regular review of the system’s operation ensures that the scheme is meeting its objectives. The review should include baseline data, performance indicators and timeframes to evaluate effectiveness [27]	Review or ‘evaluation’ is important to determine if the objective is being met [30, 32]. Review annually and at contract completion, with data obtained from monitoring informing the review process [29, 33, 39, 40, 44] In addition, seek feedback from stakeholders on strengths/weaknesses and value [32]. The review process can be used as a tool for stakeholder engagement [32]. It should also be transparent and used to communicate information about the initiative to leadership, the organisation and/or the community [32, 33]

^#^a vending skin or vending wrap refers to the vinyl wrapping around a vending machine which displays a brand or imagery (which can be negotiated in the contract terms)

## Discussion

We applied an established public health regulatory framework to publicly available guidance documents on the creation and implementation of healthy vending initiatives in order to systematically identify and create best practice recommendations. These recommendations capture the importance of both the nutrition standards and regulatory governance aspects of healthy vending to guide best practice governance mechanisms for administration, implementation, monitoring, enforcement and review when using contracts to enable effective and sustained change. This novel research fills a practice-gap for the effective use of contracts to implement healthy vending initiatives.

The documents included in this paper all focussed on healthy vending initiatives to guide organisations implementing healthy vending. These provided a a range of different perspectives, including how to create and/or implement specific healthy vending *policies*; how to create and implement healthy vending *contracts*; and how to implement healthy vending *initiatives* more broadly. The different perspectives captured in these documents add depth to our best practice recommendations, as they provide a mix of legal, public health, policy and implementation perspectives on regulatory form, substance and governance. However, in line with our previous findings, we found there was guidance encouraging organisations to implement healthy vending initiatives, but a lack of consistent information on how to maximise the chances of successful and sustained implementation, hence the significance of our best practice recommendations which provide examples of best practice regulatory form, substance and governance applied specifically to healthy food and drink vending environments [21].

### Regulatory form

Whilst our analysis identified documents from the US only, it is important for practitioners to note the local context and jurisdiction within which any initiative is implemented [25]. Healthy vending initiatives that use contracts must comply with local laws and policy contexts. Local stakeholders, including contract managers, procurement and legal counsel, should be consulted in the planning stages of any such initiative to understand the local regulatory environment and to ensure consistency with local policies.

Mandatory regulation has been considered the gold-standard for public health regulation given that voluntary or self-regulatory initiatives can be ineffective and lacking in effective monitoring and enforcement measures [45]. Only one document in our analysis explicitly discussed the need for mandatory regulation to strengthen compliance and enable enforcement measures, although others related to the use of binding contracts [29]. One document notes that although legislative action and executive orders have been used to implement healthy vending, many government sites have done so with voluntary action, indicating that although mandatory regulation is considered optimal, healthy vending initiatives have been implemented successfully without it [29].

It is possible that the use of contracts could be seen as a mandatory or enforceable regulatory measure, therefore despite there being no explicit discussion of mandatory regulation in the documents providing contractual guidance, it may have been implied in these instances [35, 42]. However, we would still argue that whilst a contract provides a means for mandatory implementation, for that implementation to be effective and sustained the contract should also include clear provisions for adequate monitoring, enforcement and review that ensure the organisation can govern the initiative effectively, as provided for in two contractual guidance documents included in the study.

Given current debates on the use of voluntary versus mandatory regulation in areas of public health – and its impact of freedom of choice—it is possible recommendations on mandatory implementation may have been excluded in most of the documents to avoid creating additional barriers to change [46, 47]. Whilst voluntary measures may not be as effective as mandatory ones, they may reduce the loss of public support while still creating a momentum for change [26, 46].

### Regulatory substance

Clearly stating the contract objective(s) ensures a healthy vending contract is clear in its intent and measurable for monitoring and review purposes [27]. Ultimately, a clear objective allows for the contract to be reviewed and judged as successful, or otherwise [25].

The operative terms and conditions form a large part of healthy vending contractual best practice. We note that some of the guidance documents identified stipulate lists of ‘allowable’ products or reference to nutrition standards. Lists of allowable products provide direct guidance to businesses who may be unfamiliar with healthy vending initiatives or the specific nutrition standards that govern the jurisdiction. They are convenient for the vending business but limited by their prescriptive nature. Prescriptive lists of food and beverages need constant revisions to ensure they include all allowable food and beverage items that meet the relevant nutrition standards. Our best practice recommendations include a suggestion from the guidance documents that reference is made in contracts to separate evidence-based nutrition standards appropriate to the jurisdiction as this allows for new products that meet those nutrition standards to be included without revisions to the contract. It also allows for revisions to the nutrition standards themselves, without alterations to the contract.

Important operative terms and conditions to clearly state in contractual documents include: guidance on preferential (equal or lower) pricing of healthy products; limits on promotion of unhealthy products, and allowable promotion on healthy products; the placement of healthier products in the high-selling positions to maximise sales; and clear labelling of healthier products visible from outside the machine. We also included a recommendation from the guidance documents that contract terms be short (less than 5 years), as this allow for market competitive conditions, and thus is considered best practice [29, 35, 42]. Longer contracts may appear competitive at the time of commencement, however shorter terms enable regular revision of contractual terms, or a change in vendor. Some jurisdictions or public agencies may have their own stipulations regarding contract length and probity, which again reinforces the importance of practitioners being aware of the local context and jurisdiction.

### Regulatory governance

Our recommendations bring attention to the need to consider all dimensions of regulatory governance in addition to the substantive terms of initiatives, beginning with the drafting of healthy vending policies or contracts. Best practice governance recommends that organisations should engage broadly with diverse stakeholders when drafting regulation [27]. We note the NANA vending guidelines were formed in consultation with a working group of approximately 40 NANA members, all with specific food and nutrition expertise [41]. This document details food and drink nutrition standards, but it also provides implementation guidance, price, placement, promotion and labelling guidance, and a rationale for healthier food and drink vending. Regulatory theory holds that consultation with a broad stakeholder group representing people from all affected parties ensures that the regulation is designed to be responsive to diverse values [9, 48]. Using the example of healthy vending, interested parties may include employees, community members, and staff representatives from human resources or wellbeing/procurement/contract and/or facilities management, unions, food and beverage vendors, and health insurance providers [33]. Broadly consulting with diverse stakeholders may also increase early support for the initiative and capture different perspectives that are important for successful and sustained implementation. However, we would caution that interested parties from the food and beverage industry may have unresolvable conflicts of interest given the profits associated with the sale of unhealthy food and drinks [49].

### Strengths/Weaknesses

We endeavoured to bring a practical application to this paper by combining healthy food initiatives with best practice regulatory governance using an established public health regulatory framework. This framework helped to break down the components of a successful contract to elucidate the key points required for the effective implementation of contracts involving a healthy food vending initiative. Our best practice recommendations have the potential to be applied to other healthy food retail initiatives governed by contracts, such as those in food retail more broadly, however further research to test the applicability of the best practice recommendations against such initiatives, is needed.

The main weakness of this study was that the guidelines identified were all produced in the US. Whilst each document references a specific or general jurisdiction in the US, our best practice recommendations acknowledged this limitation and used broader language and recommendations for best practice application to a global audience. Our sample was generated from a US seed sample where snowball sampling did not result in documents beyond the US. The relevance of the best practice recommendations to other jurisdictions requires further investigation.

We note that the commercial sensitivities of contracts limit their accessibility and that this aspect fundamentally limits their accessibility and analysis by researchers, as well as knowledge acquisition. Accordingly, our analysis, which synthesised recommendations from publicly available (online) documents helps to fill an important knowledge gap for the use of contracts to create healthier for retail environments. To our knowledge, the transition away from energy dense, high fat, sugar and salt ultra-processed foods in vending is often to foods that are less energy dense, lower in fat, sugar and salt but still ultra-processed. In this way, these foods often meet nutrition standards for vended products but are still ultra-processed, shelf stable, and easy to vend. We note this paradox given the current concerns regarding the healthiness of ultra-processed foods [50]. However, this study sought to examine the contractual mechanism used to implement and govern these initiatives, and it did not explore the merits of the nutrition standards mentioned in the guidance documents.

## Conclusion

Our study contributes to the scant literature about the use of contracts to create healthy food retail initiatives. Paying greater attention to the form, substance and governance of contracts has the potential to strengthen healthy food retail initiatives and create the conditions for their sustained implementation. Given the high rates of non-compliance seen with healthy food retail initiatives [15, 51], well-designed contracts may provide a means to improve compliance and contribute to sustained success. Our research demonstrates that nutrition standards used in healthy vending are just one component of a healthy vending initiative, with literature from the fields of regulatory studies and public health law informing our recommendations for how to create a healthy vending environment when using a contract. The regulatory governance processes of implementation, administration, monitoring, enforcement and review still remain a less studied aspect of public health nutrition interventions, and the best practice recommendations presented in Table 3 may help to bridge this gap between public health law and public health nutrition, as well as guiding the creation of future initiatives using contracts to create healthy vending, or other healthy food retail environments.

## Supplementary Information

Supplementary Material 1.

## Data Availability

Data is provided within the manuscript or supplementary information files.
